# Life-Threatening Biliary Peritonitis Following T-tube Removal: A Case Report and Literature Review

**DOI:** 10.7759/cureus.99181

**Published:** 2025-12-14

**Authors:** Lu Men, Guangbin Chen, Ke Wang, Yu Zhang, Zhilin Wang, Yiwei Li, Zhigang Liu

**Affiliations:** 1 Graduate School, Bengbu Medical University, Bengbu, CHN; 2 Department of Hepato-Pancreato-Biliary Surgery, The Second People's Hospital of Wuhu, Wuhu Hospital Affiliated to East China Normal University, Wuhu, CHN; 3 Graduate School, Wannan Medical College, Wuhu, CHN; 4 Department of Ultrasound Medicine, The Second People's Hospital of Wuhu, Wuhu Hospital Affiliated to East China Normal University, Wuhu, CHN

**Keywords:** bile ducts, biliary fistula, extrahepatic, laparoscopy, malnutrition, peritonitis, postoperative complications

## Abstract

Biliary peritonitis following T-tube removal represents a rare but potentially life-threatening complication of common bile duct exploration that underscores the critical importance of risk assessment and early intervention in T-tube management. This case report describes a 72-year-old severely malnourished male patient (body mass index (BMI) 18.1 kg/m²) with multiple comorbidities who underwent laparoscopic cholecystectomy with concomitant laparoscopic common bile duct exploration and T-tube placement for choledocholithiasis. Choledochoscopic findings at postoperative day 63 revealed incomplete fistulous tract formation at the time of T-tube removal, following which the patient developed acute and severe abdominal pain within 30 minutes, accompanied by elevated inflammatory markers. Despite aggressive conservative management, including broad-spectrum antibiotics and percutaneous drainage (800 mL of bile-stained fluid), the patient's clinical condition rapidly deteriorated to septic shock and respiratory failure within 48 hours. Emergency surgical intervention identified a 0.5×0.5 cm common bile duct fistula with extensive peritoneal bile accumulation, which was successfully repaired with new T-tube placement. Following intensive critical care management, the patient achieved complete recovery with hospital discharge after 41 days, and subsequent T-tube removal was uncomplicated. Twelve-month clinical follow-up demonstrated excellent functional outcomes with the restoration of normal appetite and absence of abdominal symptoms. This case emphasizes malnutrition as a critical and modifiable risk factor for inadequate fistulous tract formation and subsequent biliary peritonitis, highlighting the essential role of early clinical recognition, appropriate risk stratification, and coordinated multidisciplinary care with timely surgical intervention in optimizing patient outcomes.

## Introduction

Common bile duct stones (CBDS) are a frequent and potentially serious complication of gallstone disease [1]. Laparoscopic cholecystectomy combined with laparoscopic common bile duct exploration (LC+LCBDE) has emerged as the preferred single-stage surgical approach for managing gallstones with concomitant CBDS [2]. This technique offers distinct advantages including the preservation of sphincter of Oddi function, the prevention of structural biliary tract alterations, and superior stone clearance rates compared to sequential endoscopic approaches [3].

However, biliary peritonitis following T-tube removal, although uncommon with reported incidences ranging from 2.5% to 19.6%, represents a severe complication that can progress rapidly to septic shock and multi-organ failure [4]. Mortality rates range from 0% to 1.8%, with surgical reintervention required in approximately 3.1% of cases [5]. The rarity of this complication, particularly in malnourished patients, has resulted in limited literature addressing optimal management strategies and risk stratification.

This case report aims to address this knowledge gap by presenting a rare instance of life-threatening biliary peritonitis in a severely malnourished patient, emphasizing the critical role of nutritional status in fistulous tract formation and highlighting the importance of multidisciplinary management in achieving successful outcomes.

## Case presentation

Clinical presentation

A 72-year-old male patient with a medical history significant for gallstone pancreatitis, hepatitis B virus (HBV) carrier status, pulmonary tuberculosis, and prior endoscopic submucosal dissection for early gastric cancer presented with a two-month history of persistent upper abdominal discomfort and distention. The pain was characterized as dull and aching, radiating to the lumbar and dorsal regions. Physical examination revealed an afebrile, non-icteric patient with a soft, non-tender abdomen. Notably, the patient was severely malnourished with a body mass index (BMI) of 18.1 kg/m² (height: 182 cm; weight: 60 kg).

**Figure 1:**
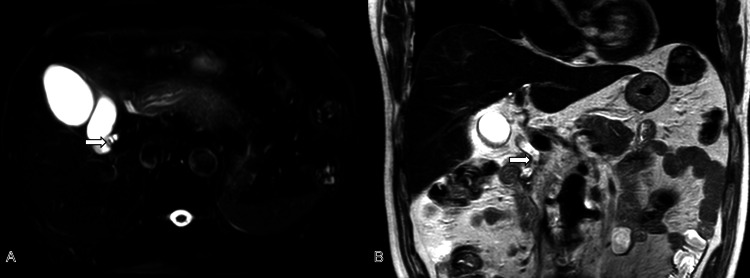
Preoperative MRCP revealed small stones in the cystic duct (white arrow; A) and multiple stones in the common bile duct (white arrow; B). MRCP: magnetic resonance cholangiopancreatography

Laboratory investigations demonstrated normal hepatic and renal function parameters, with serum electrolytes and amylase levels within normal limits (Table 1). Abdominal ultrasonography revealed gallbladder wall thickening with hyperechoic foci in the neck region. Magnetic resonance cholangiopancreatography (MRCP) identified small hepatic cysts, sludge-like gallbladder stones, cholecystitis, possible sludge-like CBDS, and evidence of resolved acute pancreatitis (Figure 1).

**Table 1:** Patient baseline characteristics and preoperative laboratory findings at initial admission. ALT: alanine aminotransferase; AST: aspartate aminotransferase; ALP: alkaline phosphatase; GGT: gamma-glutamyl transferase

Laboratory test	Day 2 of first admission	Normal reference range
ALT	7 U/L	9-50 U/L
AST	15 U/L	15-40 U/L
ALP	109 U/L	45-125 U/L
GGT	19 U/L	10-60 U/L
Total bilirubin	10.8 μmol/L	2-23.4 μmol/L
Direct bilirubin	1.9 μmol/L	0-9.8 μmol/L
Indirect bilirubin	8.9 μmol/L	1.7-17 μmol/L
Uric acid	298 μmol/L	208.3-428.4 μmol/L
Urea	6.94 mmol/L	3.6-9.5 mmol/L
Creatinine	79.2 μmol/L	57-111 μmol/L
Total protein	63.6 g/L	65-85 g/L
Albumin	37.8 g/L	40-55 g/L

Investigations and initial treatment

Based on the clinical presentation and imaging findings, the patient was diagnosed with choledocholithiasis and cholecystolithiasis with cholecystitis. Differential diagnoses including malignancy, stricture, and infectious causes were excluded based on imaging characteristics and laboratory parameters. The patient subsequently underwent LC+LCBDE. Intraoperatively, the gallbladder appeared congested and edematous. Choledochoscopy confirmed multiple stones in the mid-to-lower bile duct, with the largest measuring approximately 20×16 mm. Following complete stone extraction, an 18-French latex T-tube was placed in the common bile duct prior to procedure completion.

The patient's initial postoperative recovery was uncomplicated. Abdominal ultrasonography on postoperative day 7 demonstrated no intraperitoneal or pelvic fluid collections, with the T-tube in the appropriate position. The patient was discharged home the same day.

Complications and clinical deterioration

On postoperative day 63, the patient returned for elective T-tube removal. During the procedure, no bile leakage was observed following T-tube extraction. However, choledochoscopy revealed incomplete fistulous tract formation. Given this finding, an abdominal drainage catheter was placed to facilitate bile drainage, prevent fluid accumulation and infection in the peritoneal cavity, and allow closer monitoring of the patient's condition to reduce post-T-tube removal complications.

**Figure 2:**
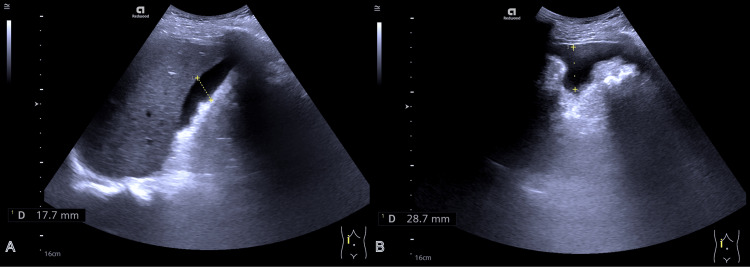
Ultrasound exploration of the abdomen detected a small amount of fluid around the liver (dotted line; A-B).

Within 30 minutes of T-tube removal, the patient developed severe, persistent abdominal pain. Physical examination revealed right upper quadrant tenderness without rebound tenderness or abdominal rigidity. Laboratory studies showed leukocytosis with neutrophilia (Table 2). Abdominal ultrasonography detected a small volume of intraperitoneal fluid, consistent with biliary peritonitis (Figure 2). Despite the initiation of broad-spectrum antibiotics and intravenous fluid resuscitation, symptoms failed to improve significantly.

**Table 2:** Serial changes in inflammatory markers following T-tube removal. WBC: white blood cell; IL-6: interleukin-6; CRP: C-reactive protein

Laboratory parameters	Day 1 after T-tube removal and bile leak (2nd admission, day 1)	Day 2 after T-tube removal and bile leak (2nd admission, day 2)	Day 4 after emergency surgery (2nd admission, day 7)	Normal reference range
WBC count	12.42×10⁹/L	5.27×10⁹/L	11.02×10⁹/L	3.5-9.5×10⁹/L
Neutrophil	89%	80%	79.2%	40-75%
Hemoglobin	146 g/L	146 g/L	110 g/L	130-175 g/L
Platelet count	212×10⁹/L	190×10⁹/L	109×10⁹/L	125-350×10⁹/L
Total bilirubin	33.6 μmol/L	18.8 μmol/L	16.3 μmol/L	2-23.4 μmol/L
Sodium	129.8 mmol/L	134.3 mmol/L	141.7 mmol/L	137-147 mmol/L
Potassium	3.78 mmol/L	6.22 mmol/L	4.82 mmol/L	3.5-5.3 mmol/L
Carbon dioxide	23.2 mmol/L	16.5 mmol/L	22 mmol/L	22-32 mmol/L
Amylase	66 U/L	180 U/L	70 U/L	35-135 U/L
IL-6	>5000 pg/mL	>5000 pg/mL	30.6 pg/mL	≤7 pg/mL
Procalcitonin	3.99 ng/mL	54.3 ng/mL	29.7 ng/mL	≤0.5 ng/mL
CRP	131 mg/L	357 mg/L	61.1 mg/L	0-8 mg/L

Over the subsequent 24 hours, inflammatory markers deteriorated markedly, with C-reactive protein rising from 131 to 357 mg/L and interleukin-6 levels increasing dramatically (Table 2), accompanied by the progressive accumulation of intraperitoneal fluid. Ultrasound-guided percutaneous abdominal paracentesis was performed, yielding 800 mL of bile-stained fluid, providing temporary symptomatic relief.

Emergency management and recovery

On postoperative day 65, the patient's condition deteriorated precipitously, necessitating intensive care unit (ICU) admission for the management of septic shock and acute respiratory failure. Despite mechanical ventilation and high-dose vasopressor support, hemodynamic instability persisted. Bedside ultrasonography revealed extensive free fluid collections around the liver and within the pelvis, with a maximum depth of approximately 28 mm. Following multidisciplinary consultation, emergency surgical exploration was immediately undertaken.

Emergency laparotomy revealed a 0.5×0.5 cm fistula on the common bile duct surface with extensive bile accumulation throughout the peritoneal cavity. Following thorough aspiration and copious warm saline irrigation of the peritoneal cavity, the choledochal fistula was debrided and repaired. A new 18-French latex T-tube was inserted and secured with sutures to ensure optimal positioning and stability.

Postoperatively, the patient remained in the ICU receiving intensive antimicrobial therapy and comprehensive nutritional support. His clinical condition improved progressively (Table 2), allowing transfer to the general ward on postoperative day 69. Subsequently, the patient developed recurrent vomiting, which was diagnosed as gastroparesis following appropriate investigations. This complication was successfully managed with gastrointestinal decompression, nutritional optimization, and adjunctive traditional Chinese medicine acupuncture therapy.

**Figure 3:**
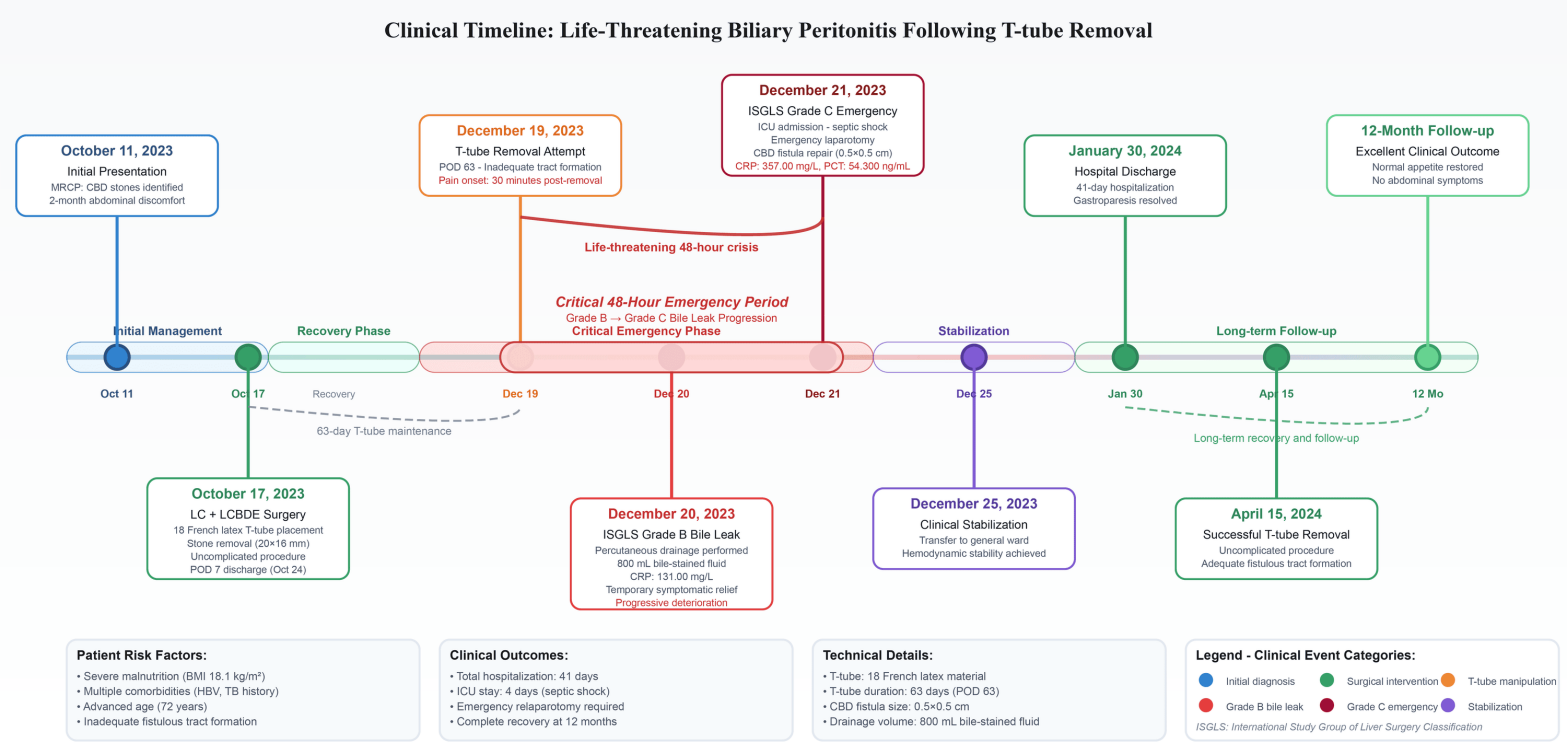
Complete clinical timeline of biliary peritonitis following T-tube removal. The timeline demonstrates the critical 48-hour period during which the patient transitioned from routine T-tube removal to life-threatening septic shock, highlighting key temporal relationships and multidisciplinary interventions that led to successful clinical recovery. MRCP: magnetic resonance cholangiopancreatography; CBD: common bile duct; LC+LCBDE: laparoscopic cholecystectomy combined with laparoscopic common bile duct exploration; ISGLS: International Study Group of Liver Surgery; CRP: C-reactive protein; ICU: intensive care unit; BMI: body mass index; HBV: hepatitis B virus; PCT: procalcitonin Image Credits: Guangbin Chen

The patient achieved complete clinical recovery and was discharged after a 41-day hospitalization. T-tube removal was successfully performed without complications four months later. At the 12-month follow-up, the patient demonstrated excellent clinical outcomes with normal appetite and the absence of abdominal pain or distention (Figure 3).

## Discussion

Pathophysiology and risk assessment

The pathophysiological foundation of successful T-tube management depends on the formation of a mature fibrous bilio-cutaneous fistulous tract [6]. This tract develops through inflammatory changes induced by the physical presence of the T-tube, with the inflammatory cascade primarily mediated by lymphocytes, plasma cells, and histiocytes orchestrating structured fibrous encapsulation [7]. Adequate tract maturation is essential for preventing bile extravasation upon T-tube removal. Insufficient tract formation can result in bile leakage into the peritoneal cavity, triggering chemical peritonitis that may rapidly progress to bacterial peritonitis, sepsis, and multi-organ failure [8].

Our case highlights several critical risk factors that significantly increase the likelihood of this complication. Malnutrition represents a critical and potentially modifiable risk factor that significantly impairs wound healing and delays fistulous tract formation. Our patient demonstrated severe malnutrition with a BMI of 18.1 kg/m², which likely compromised the inflammatory response necessary for robust tract formation [9]. Protein deficiency particularly affects collagen synthesis and immune function, emphasizing the importance of preoperative nutritional assessment and optimization [10].

Management strategy and clinical application

The International Study Group of Liver Surgery (ISGLS) bile leak grading system provides invaluable guidance for management decisions, stratifying leaks based on severity and required interventions [11]. This case illustrates the dynamic nature of bile leak severity and underscores the importance of serial clinical and laboratory assessments. Our patient initially presented with a Grade B bile leak, which rapidly progressed to Grade C status within 24 hours, necessitating emergency surgical intervention.

The rapid progression from initial symptoms to life-threatening septic shock highlights the potential for abrupt clinical deterioration in elderly, malnourished patients with biliary peritonitis.

The successful management of our patient underscores the paramount importance of multidisciplinary collaboration in addressing complex surgical complications. Integration of perspectives from hepatobiliary surgery, critical care medicine, interventional radiology, and infectious disease specialists facilitated comprehensive evaluation and coordinated intervention [12].

Clinical implications and prevention strategies

This case highlights several important considerations for clinical practice. Comprehensive nutritional assessment using validated screening tools should be implemented for all patients scheduled for biliary procedures, with high-risk patients receiving nutritional optimization when clinically feasible [13]. T-tube material choice should be tailored to patient characteristics, with the consideration of latex T-tubes for higher-risk patients, balancing enhanced inflammatory response benefits against increased tissue reactivity risks [14]. T-tube placement duration should be extended beyond conventional timeframes for malnourished patients, and we advocate for fistulography prior to removal in high-risk patients to confirm adequate tract formation [15].

## Conclusions

Biliary peritonitis following T-tube removal represents a rare but potentially life-threatening complication. This case report demonstrates that severe malnutrition significantly impairs bile duct fistulous tract formation and increases complication risk. Early recognition of incomplete tract formation through choledochoscopy or fistulography, appropriate risk stratification based on nutritional status, and vigilant postoperative monitoring are essential for safe T-tube management. When biliary peritonitis occurs, aggressive multidisciplinary management integrating surgical expertise, critical care support, and comprehensive nutritional therapy is crucial for favorable outcomes. Through careful preoperative nutritional assessment, individualized T-tube retention strategies, objective confirmation of adequate tract maturation, and prompt intervention when complications arise, clinicians can minimize this serious complication and optimize patient outcomes.
